# Sterile Milk for Infant Feeding in France

**Published:** 1903-05-09

**Authors:** 


### STERILE MILK FOR INFANT FEEDING IN FRANCE.

Doctor Rittebrand [1] reports favourably on some French institutions which have for their object the distribution of sterilised milk for infants. In France, where the populace has shown a steady decrease during the last decades, it is most essential to counteract this tendency in every possible quarter. Infant mortality is one of the items that weighs heavily on a nation's death-rate, and this infant mortality is to a great extent dependent upon the method of infant feeding. Urged by this a number of French medical men have founded various institutions during the last ten years, with the purpose of enabling large circles to get sterile milk cheaply and easily, and have so endeavoured to cause a decrease in the enormous infant mortality of the nation, especially in large towns and industrial centres. Dr. Budin founded the first establishment of this kind by opening a polyclinic for infants in Paris, where poor mothers who were not themselves able to feed their babies received sterilised milk gratuitously. His example was followed by 20 metropolitan hospitals. One of these stations for the supply of sterile milk, in the Rue Oudinot, distributes sterilised milk daily at 10 a.m. The price is put down at 30 centimes a litre, but poor mothers receive it free of cost, if they can produce satisfactory testimonials as to their needy condition from the parish authorities. Once a week the infants have to be brought to the polyclinic, in order to be inspected by the physician and weighed. Sick children receive treatment at the polyclinic free of cost. The milk is supplied every morning except on Sundays and other holidays, when a double ration is given on the previous day. All milk must be paid for at once. Ten centimes is charged for the glass bottle, and refunded when this is returned. All the necessary utensils, teats, brushes, etc., are supplied free of cost, and renewed when required, but only if the old damaged articles are brought back to avoid abuse. The mothers are entitled to the benefits of the institution, as long as they adhere to the few rules, but forfeit all claim immediately they disobey them. The results, according to statistics, are excellent: during 1900 not one of the 422 Rue Oudinot infants died of gastro-enteritis and Dr. Bresset shows how wonderfully infant mortality has decreased in the whole metropolis since the systematic distribution of sterile milk has been instituted. The mortality of infants from gastro-enteritis amounted to 3,274 in 1896; in 1900 it stood at 2,461, an improvement of 24 per cent. The total mortality of infants from gastro-enteritis during the years 1891 to 1895 inclusive was 15,014; in the years 1896 to 1900 the total mortality from this cause was 13,355, an improvement of 11 per cent. Another enterprise, the gouttes de lait—drops of milk—is even more likely to lead to good results. These gouttes de lait supply each individual feed, ready mixed and sterilised, in small feeding-bottles. The mother receives as many of these bottles every morning as the infant will require feeds during the 24 hours. The first establishment of this kind was started by Dufour at Fécamp, and about ninety towns followed suit almost immediately. The Gouttes de lait in Versailles, founded by Dr. Broussin is favourably known for being managed on most practical and economical principles. The staff consists of a steward who looks after the business part and the distribution of the milk, and a maid who sterilises the milk and cleans the utensils. Several doctors and lay persons supervise the establishment. The milk is milked on the farms at 5.30 a.m., arrives at the office at 6.30, and is immediately filled into the portion bottles, and diluted with water; sugar and salt are added to each bottle which is lightly closed by laying on a disk of india-rubber, and then the mixture is exposed to a heat of 102 C. for three quarters of an hour. The mother receives a wire basket with the requisite number of feeds every morning, and returns another basket with empty bottles at the same time. The scale of payment varies with the class of patients. For the poor it is 20 centimes a day, for the middle-class 50 centimes, and for the wealthy 1 franc. Broken bottles must be paid for at once. Statistics are kept most carefully about every child. The weekly weighing results are entered on charts with a normal increase curve, and in case of loss of weight the doctor steps in and alters the diet, etc. This weekly inspection also guarantees to a certain extent the children being kept clean and tidy. The great advantage of these gouttes de lait is the ease with which the mother can feed the child after simply standing the bottle in which she receives the infant's portion in hot water. There is no danger of dirt getting into the milk, or a wrong mixture being prepared, as when she receives the one large bottle of undiluted sterilised milk spoken of before.

[1] Die Krankenpflege, Jahrgang 2, Heft. I.

